# Next-generation sequencing reveals mitogenome diversity in plasma extracellular vesicles from colorectal cancer patients

**DOI:** 10.1186/s12885-023-11092-x

**Published:** 2023-07-12

**Authors:** Tonje Bjørnetrø, Paula A. Bousquet, Kathrine Røe Redalen, Anne-Marie Siebke Trøseid, Torben Lüders, Espen Stang, Adriana M. Sanabria, Christin Johansen, Anniken Jørlo Fuglestad, Christian Kersten, Sebastian Meltzer, Anne Hansen Ree

**Affiliations:** 1 Department of Oncology, Akershus University Hospital, P.O. Box 1000, 1478 Lørenskog, Norway; 2 Department of Physics, Norwegian University of Science and Technology, Trondheim, Norway; 3 Department of Biochemistry, Oslo University Hospital Ullevål, Oslo, Norway; 4 Department of Clinical Molecular Biology, Akershus University Hospital, Lørenskog, Norway; 5 Institute of Clinical Medicine, University of Oslo, Oslo, Norway; 6 Department of Pathology, Oslo University Hospital Rikshospitalet, Oslo, Norway; 7 Department of Research, Southern Hospital Trust, Kristiansand, Norway

**Keywords:** mtDNA, Next-generation sequencing, Extracellular vesicles, Colorectal cancer

## Abstract

**Background:**

Recent reports have demonstrated that the entire mitochondrial genome can be secreted in extracellular vesicles (EVs), but the biological attributes of this cell-free mitochondrial DNA (mtDNA) remain insufficiently understood. We used next-generation sequencing to compare plasma EV-derived mtDNA to that of whole blood (WB), peripheral blood mononuclear cells (PBMCs), and formalin-fixed paraffin-embedded (FFPE) tumor tissue from eight rectal cancer patients and WB and fresh-frozen (FF) tumor tissue from eight colon cancer patients.

**Methods:**

Total DNA was isolated before the mtDNA was enriched by PCR with either two primer sets generating two long products or multiple primer sets (for the FFPE tumors), prior to the sequencing. mtDNA diversity was assessed as the total variant number, level of heteroplasmy (mutant mtDNA copies mixed with wild-type copies), variant distribution within the protein-coding genes, and the predicted functional effect of the variants in the different sample types. Differences between groups were compared by paired Student’s *t*-test or ANOVA with Dunnett’s multiple comparison tests when comparing matched samples from patients. Mann–Whitney U test was used when comparing differences between the cancer types and patient groups. Pearson correlation analysis was performed.

**Results:**

In both cancer types, EV mtDNA presented twice as many variants and had significantly more low-level heteroplasmy than WB mtDNA. The EV mtDNA variants were clustered in the coding regions, and the proportion of EV mtDNA variants that were missense mutations (i.e., estimated to moderately affect the mitochondrial protein function) was significantly higher than in WB and tumor tissues. Nonsense mutations (i.e., estimated to highly affect the mitochondrial protein function) were only observed in the tumor tissues and EVs.

**Conclusion:**

Taken together, plasma EV mtDNA in CRC patients exhibits a high degree of diversity.

**Trial registration:**

ClinicalTrials.gov: NCT01816607. Registered 22 March 2013.

**Supplementary Information:**

The online version contains supplementary material available at 10.1186/s12885-023-11092-x.

## Background

Colorectal cancer (CRC) is the third most common cancer type worldwide [1]. It is a heterogeneous disease in terms of high biological complexity and clinical outcome. Extracellular vesicles (EVs) are known to contribute to tumorigenesis, progression, and drug resistance in CRC [2] and may be important CRC biomarkers [3]. Mutations in mitochondrial genes have been reported to have a role in cancer development [4]. Variations in the mitochondrial DNA (mtDNA) sequence can act as functional adaptors allowing tumor and immune cells to adjust to the metabolic needs imposed by various tissue environments during cancer progression [5]. Recent reports have demonstrated that the entire mitochondrial genome can be packed inside EVs [6, 7] and restore metabolic activity in cells with impaired metabolism [6].

The mitochondrial genome is a 16.5-kilobase circular double-stranded DNA molecule present in multiple copies per cell. It contains 37 genes that encode 13 protein subunits of the mitochondrial respiratory chain/oxidative phosphorylation system, two rRNAs, and 22 tRNAs for mitochondrial translation [8]. The mtDNA replication is independent of the cell cycle and also occurs in postmitotic cells. Because the mutation frequency of replicating mtDNA is high, mutant mtDNA copies are often mixed with wild-type copies in the cell (termed heteroplasmy). The mtDNA polymorphisms may alter mitochondrial function, particularly in tissues that are highly dependent on the metabolism. Nevertheless, if a mutation is pathogenic, the cell can often tolerate a certain proportion of the mtDNA variant before the biochemical threshold is exceeded with resulting metabolic defects [8].

Cell-derived mitochondrial components, besides mtDNA, have been found in the extracellular space [9]. For example, secreted cell-free respiratory-competent mitochondria have been detected in blood [10] and EVs have been shown to contain functional mitochondria [11] and be enriched in mitochondrial proteins [12]. In addition, a novel EV population of mitochondrial origin, mitovesicles, was described by D’Acunzo et al*.* [13].

Despite the increasing interest in EV mitochondrial components, the characteristics of secreted cell-free mtDNA still remain insufficiently understood. Here, we present a method for the successful isolation and sequencing of the full mitochondrial genome from whole blood (WB), peripheral blood mononuclear cells (PBMCs), plasma EVs, and tumor tissue from CRC patients as an initial investigation for the potential use of EVs as a source of cell-free mtDNA and their potential as CRC biomarker. We have further analyzed mtDNA diversity by assessing the total variant number, level of heteroplasmy, variant distribution within the protein-coding genes, and the predicted functional effect of the variants in the different sample types.

## Methods

### Patients and procedures

The rectal cancer patients were enrolled onto a prospective biomarker study (ClinicalTrials.gov: NCT01816607) conducted at Akershus University Hospital (Lørenskog, Norway) and the colon cancer patients participated in a prospectively maintained CRC database and ancillary biobank at Southern Hospital Trust (Kristiansand, Norway). All patients had histologically verified colon or rectal adenocarcinoma without metastatic disease at the time of diagnosis, but the rectal cancer patients presented tumor manifestations within the pelvic cavity that were considered at high risk of disease recurrence and were consequently given chemoradiotherapy before the surgical procedure. All patients received curative-intent treatment according to prevailing national guidelines. The patient and disease characteristics are shown in Supplementary Table S1. The total group of patients was representative for the distribution of men and women affected by CRC.

### Preparation of patient samples

In this study, we included various biospecimens from CRC patients. Each of eight rectal cancer patients provided WB, PBMCs, citrate plasma, and formalin-fixed paraffin-embedded (FFPE) tumor tissue that were sampled at the time of diagnosis, and stored for a median of 23 (range, 10–37), 47 (range, 34–62), 50 (range, 35–64), and 53 (range, 40–69) months, respectively. Each of eight colon cancer patients provided WB at the time of diagnosis (stored for a median of 78 (range, 68–85) months) and fresh-frozen (FF) tumor tissue sampled within an hour after rigorous standard operating procedure (stored for a median of 75 (range, 68–83) months from the surgical resection).

The tumor tissues were cut in 30-μm sections of 25–100 mm^2^ tissue with at least 20% tumor cells, as determined by an experienced specialist in gastrointestinal pathology, prior to analysis. The WB was collected by venipuncture in sodium citrate-treated BD Vacutainer CPT tubes (Becton, Dickinson and Company, Franklin Lakes, NJ, USA) for preparation of PBMCs and PAXgene RNA tubes (PreAnalytiX GmbH, Hombrechtikon, Switzerland), the latter stored at –80 °C until analysis. Citrate plasma samples were prepared by centrifugation at 2,000* g* for 10 min, and aliquots were stored at –80 °C. The PBMC specimens were prepared from 6–8 ml of WB by centrifugation with a horizontal rotor centrifuge at 1500* g* for 20 min. The buffy coat layer was transferred to a fresh 15-ml tube, resuspended and washed twice in phosphate-buffered saline (Gibco by Life Technologies, Paisley, UK) with centrifugations at 300* g* for 15 and 10 min. The mononuclear cells were thereafter resuspended in RPMI-1640 medium (Gibco) supplemented with 10% dimethyl sulfoxide (Sigma-Aldrich, Saint Louis, MO, USA) and immediately frozen at –150 °C. Prior to DNA extraction, 150 μl of thawed PBMC preparations or PAXgene WB samples were transferred to microcentrifuge tubes and centrifuged at 5000* g* for 10 min before the supernatants were carefully removed.

### Isolation and characterization of EVs

EVs (heterogeneous populations of small and medium sized vesicles) were isolated from 100 μl plasma using qEV Single Size Exclusion Chromatography Columns (IZON Science, Oxford, UK). The columns were equilibrated with 10 ml of 0.20-μm-filtered phosphate-buffered saline. EVs were isolated after 1 ml void volume as 250-μl fractions, and the eluted fractions number 5 and 6 were combined. All samples were stored at –80 °C. The size and concentration of the vesicles were determined by Nanoparticle Tracking Analysis (NTA; Malvern, Amesbury, UK). Here, three 60-s videos were captured for each sample (slide shutter 1206 or 1259, slider gain 366) and the videos were analyzed by the NTA 3.4 software (Malvern). For morphological examination and detection of EV-associated proteins, EVs from one of the patient samples were analyzed with transmission electron microscopy (TEM) and western blot. Formvar/carbon-supported 100 mesh hexagonal copper grids (Electron Microscopy Sciences, Hatfield, PA, USA) were places on top of a 5 μl drop of the EV sample for 5 min. The grids were washed three times with distilled H_2_O before incubation with 2% methylcellulose (Sigma) containing 0.3% uranyl acetate (Electron Microscopy Sciences) for 10 min on ice. Surplus of methylcellulose-uranyl acetate was removed using a filter paper and the grids were air dried before examination using a Tecnai G^2^ Spirit TEM (FEI, Eindhoven, The Netherlands) equipped with a Morada digital camera using RADIUS imaging software. Images were processed using Adobe Photoshop. Prior to the western blot analysis, 500 μl of the EV solution was concentrated using Vivaspin® 500 10 K centrifugal concentrator (Sartorius Stedim Lab, Stonehouse, UK) and lysed in M-PER® Mammalian Protein Extraction Reagent supplemented with Halt™ Protease Inhibitor Cocktail and Halt™ Phosphotase inhibitor Cocktail (all from Thermo Fisher Scientific, Waltham, MA, USA). For detection of CD9 and CD63, non-reducing conditions were used. 10 μg protein from EVs and 5 μg from HCT116 cells (a CRC cell line as positive control) were separated by NuPAGE Bis–Tris (Novex by Life Technologies, Carlsbad, CA, USA) and transferred to Immobilon-P membranes (Millipore Corporation, Billerica, MA, USA). The primary antibodies were anti-CD9 (Ts9 1:500) and anti-CD63 (Ts63 1:500; both from Thermo Fisher Scientific), anti-ALIX (3A9 1:500; Abcam, Cambridge, UK), anti-APOA1 (B-10 1:1000; Santa Cruz Biotechnology, Heidelberg, Germany), and anti-GM130 XP (D6B1 1:1000; Cell Signaling Technology, La Jolla, CA, USA). Secondary antibodies were from Dako Denmark AS (Glostrup, Denmark). Peroxidase activity was visualized using SuperSignal West Dura Extended Duration Substrate (Thermo Fisher Scientific) and the membranes were scanned with ImageQuant Las 3000 system (FujiFilm, Tokyo, Japan). Positive bands were analyzed using Fujifilm Multi Gauge V3.1 and the images of all full-length blots are provided in the Supplementary Information file. All relevant data of our experiments has been submitted to the EV-TRACK knowledgebase (EV-TRACK ID: EV210384).

### DNase treatment of EVs

The samples and reagents were thawed on ice and 200 μl of samples were first incubated with 20 μl DNase (DNaseI Amplification Grade; Sigma-Aldrich) at 37 °C. After 30 min 40 μl Proteinase K (Qiagen, Hilden, Germany) was added and the samples were further incubated at 37 °C for 30 min before 20 μl stop-solution was added and the samples were incubated at 70 °C for 10 min. The samples were put on ice and stored at –80 °C. Specifically for the evaluation of contaminating DNA from outside of EVs, samples from two patients were pooled before 100 μl aliquots were incubated with or without DNase in triplicates.

### DNA isolation

QIAamp DNA FFPE Tissue Kit and DNeasy Blood & Tissue Kit (Qiagen) were used to extract DNA from FFPE tissues and the other tissues, respectively, according to the manufacturer’s protocols. To increase the DNA yield from the EV samples, an additional spin with open tubes was performed prior to DNA elution, and the samples were eluted with water preheated to 70 °C. For all samples, DNA was quantified using Nanodrop ND 1000 Spectrophometer and the Qubit fluorometer 2.0 in combination with the Qubit dsDNA HS Assay Kit (all from Thermo Fisher Scientific).

### mtDNA sequencing

For the WB, PBMC, EV, and FF tumor specimens, the mtDNA was amplified using two pairs of site-specific primers (forward: MTL-F1 5’- AAA GCA CAT ACC AAG GCC AC -3’and MTL-F2 5’- TAT CCG CCA TCC CAT ACA TT -3’; reverse: MTL-R1 5’- TTG GCT CTC CTT GCA AAG TT -3’ and MTL-R2 5’- AAT GTT GAG CCG TAG ATG CC -3’) and TaKaRa LA TaqDNA polymerase (TaKaRa-Bio, Kusatsu, Japan), to generate two long fragments spanning the complete mitochondrial genome. The two primer pairs failed to amplify the mtDNA in FFPE-samples and we developed an mtDNA amplification procedure based on 21 primer sets that produced overlapping PCR products to generate the complete mitochondrial genome from FFPE tissue DNA. Of these, 12 primers (Supplementary Table S2) have been published by Levin et al*.*[14] and 9 primers (Supplementary Table S3) were a combination of the published primers. The process of library preparation followed the suggested protocols of Human mtDNA Genome for the Illumina Sequencing platform (Illumina, Inc., San Diego, CA) with the first steps of the protocol adjusted. The master mixes were divided into 4 PCR tubes per sample, and a temperature gradient (51–68 °C) was used during the first amplification. The DNA was subsequently purified using gel electrophoresis, and bands representing the circular mtDNA amplicon (9.1 kilobases and 11.2 kilobases) or short PCR products were cut out from the gel. Extraction and quantification of mtDNA were performed using QIAEX II Gel Extraction Kit (Qiagen) and Qubit dsDNA HS Assay (Thermo Fisher Scientific). Successful long-range PCRs were represented by a bright band of the expected size. The amplicons were pooled and libraries were generated using the Nextera XT DNA Library Preparation Kit and Nextera XT Index Kit (both Illumina). AMPure XP beads (Beckman Coulter, Brea, CA, USA) were used to purify the DNA library and provide a size-selection step to remove short library fragments. Bioanalyzer-based normalization was performed using the Agilent High-Sensitivity DNA Kit (Agilent Technologies, Waldbronn, Germany) and the libraries were pooled and sequenced on a MiSeq Benchtop Sequencer (Illumina) using a MiSeq Reagent Kit v3 (Illumina) with 2 × 300-basepair read lengths.

### mtDNA variant analyses

All sequence data generated was mapped to the revised Cambridge Reference Sequence (GenBank ID NC_012920.1) [15, 16] using the MiSeq Reporter built-in software v2.6 (Illumina). This software applies a Burrows-Wheeler Aligner [17] and generates BAM alignment files. The Mutserve via mtDNA-Server (https://mtdna-server.uibk.ac.at) [18] was used for variant calling and annotation with default parameters and filter settings; Minimum Base Call Quality Score for a Call (< 30), Indel Repeat Length (> 8), and Low Variant Frequency (< 0.010). Only variants with final filter pass were included for downstream analysis. This variant caller has various internal quality controls and was shown to have best performance compared to other variant callers in regards to evaluating heteroplasmy [19]. Variant frequencies > 0.990 were defined as homoplasmy, while heteroplasmy was defined by frequencies of 0.10–0.990 and low-level heteroplasmy by < 0.10. Variants flagged as previously reported nuclear mitochondrial DNA (NUMTs) by Mutserve were identified. Haplocheck v1.3.2 was used to detect contamination in the mtDNA samples [20] and Haplogrep v2.3.0 for haplogroup classification [21] through the mtDNA-Server. The Ensembl Variant Effect Predictor software was used with default parameters to predict the potential role of the variants [22]. An mtDNA circular plot was made in Geneious (v2023.0).

### Quantification of mtDNA damage

This assay relies on the ability of a modification on the template DNA to inhibit restriction enzyme cleavage, as detailed previously [23]. Total DNA from the DNase-treated and non-treated pooled patient samples was analyzed with droplet digital PCR. A sequence flanking a TaqI restriction enzyme site in the 12S ribosomal RNA gene (*MT-RNR1*) was amplified using the forward (5′- AAA CTG CTC GCC AGA ACA CT -3′) and reverse (5′- CAT GGG CTA CAC CTT GAC CT-3′) primers in the absence and presence of the enzyme. The samples were partitioned by the QX200 Droplet Generator (Bio-Rad Laboratories, Oslo, Norway) and analyzed with the QX200 Droplet Reader (Bio-Rad Laboratories). The data was given as the percentage of non-digested (nd) mtDNA [(mtDNA^TaqI^ copies per μl – mtDNA^nd^ copies per μl) × 100].

### Statistical considerations

Analyses were performed using GraphPad Prism v9.2.0. Differences between groups were compared by paired Student’s *t*-test or Repeated-Measures ANOVA with Dunnett’s multiple comparison tests when comparing one variable in matched samples from patients and when comparing several variables, a 2-way ANOVA was used. Mann–Whitney U test was used when comparing differences between the cancer types and patient groups. *p*-values less than 0.05 were considered statistically significant. Pearson correlation analysis was performed.

## Results

### mtDNA variant number and heteroplasmy – rectal cancer patients

**Fig. 1:**
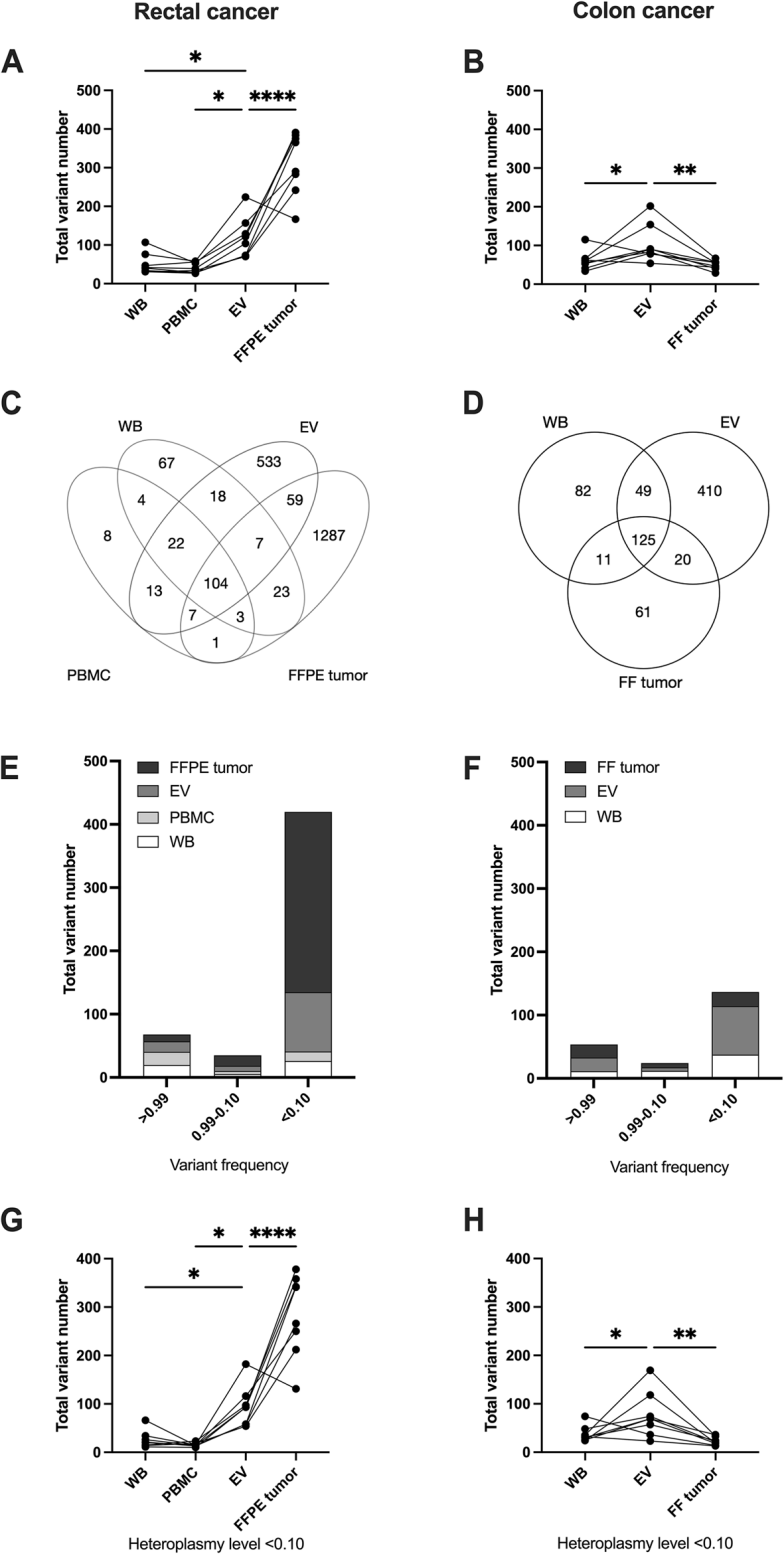
Mitochondrial DNA variant number and the level of heteroplasmy in the colorectal cancer patients. Total variant number in (**A**) whole blood (WB), peripheral blood mononuclear cells (PBMC), plasma extracellular vesicles (EV), and formalin-fixed paraffin-embedded (FFPE) tumor specimens from rectal cancer patients (*n* = 8) and in (**B**) WB, EV, and fresh-frozen (FF) tumor specimens from colon cancer patients (*n* = 8). Points connected with a line represent matched patient specimens. The mean number of all variants detected in each sample type was compared to the EV number by Dunnett´s multiple comparison. Venn diagram of all variants detected in the different sample types from (**C**) rectal (*n* = 8) and (**D**) colon (*n* = 8) cancer patients. Heteroplasmy levels in the different sample types from (**E**) rectal (*n* = 8) and (**F**) colon (*n* = 8) cancer patients. Low-level heteroplasmy (< 0.10) variants in the different sample types from (**G**) rectal (*n* = 8) and (**H**) colon (*n* = 8) cancer patients. Points connected with a line represent matched patient specimens. The mean number of all variants detected in each sample type was compared to the EV number by Dunnett´s multiple comparison. *p*-values for all relevant panels: *, 0.033; **, 0.0021; ***, 0.0002;****, < 0.0001

The median coverage depth was 15 237 × . After processing the sequences with adequate quality scores (Q30, median of 89.9%), the proportions of aligned sequence reads for WB, PBMCs, EVs, and FFPE tumor tissue were 98.7%, 99.7%, 99.8%, and 99.8%, respectively. The ratio of transversions and transitions, and GC content across the mitochondrial genomes were comparable for all four tissue specimens from the eight rectal cancer patients (Supplementary Table S4). As expected [24], the total number of variants was similar in WB and PBMCs with a median of 41.0 (range, 31–107) and 36.5 (range, 27–58), respectively (Fig. 1a). When comparing all tissue types, differences were detected (Repeated-Measures ANOVA: sample types (column) *p* < 0.0001 and patients (row) *p* = 0.71).

**Fig. 2:**
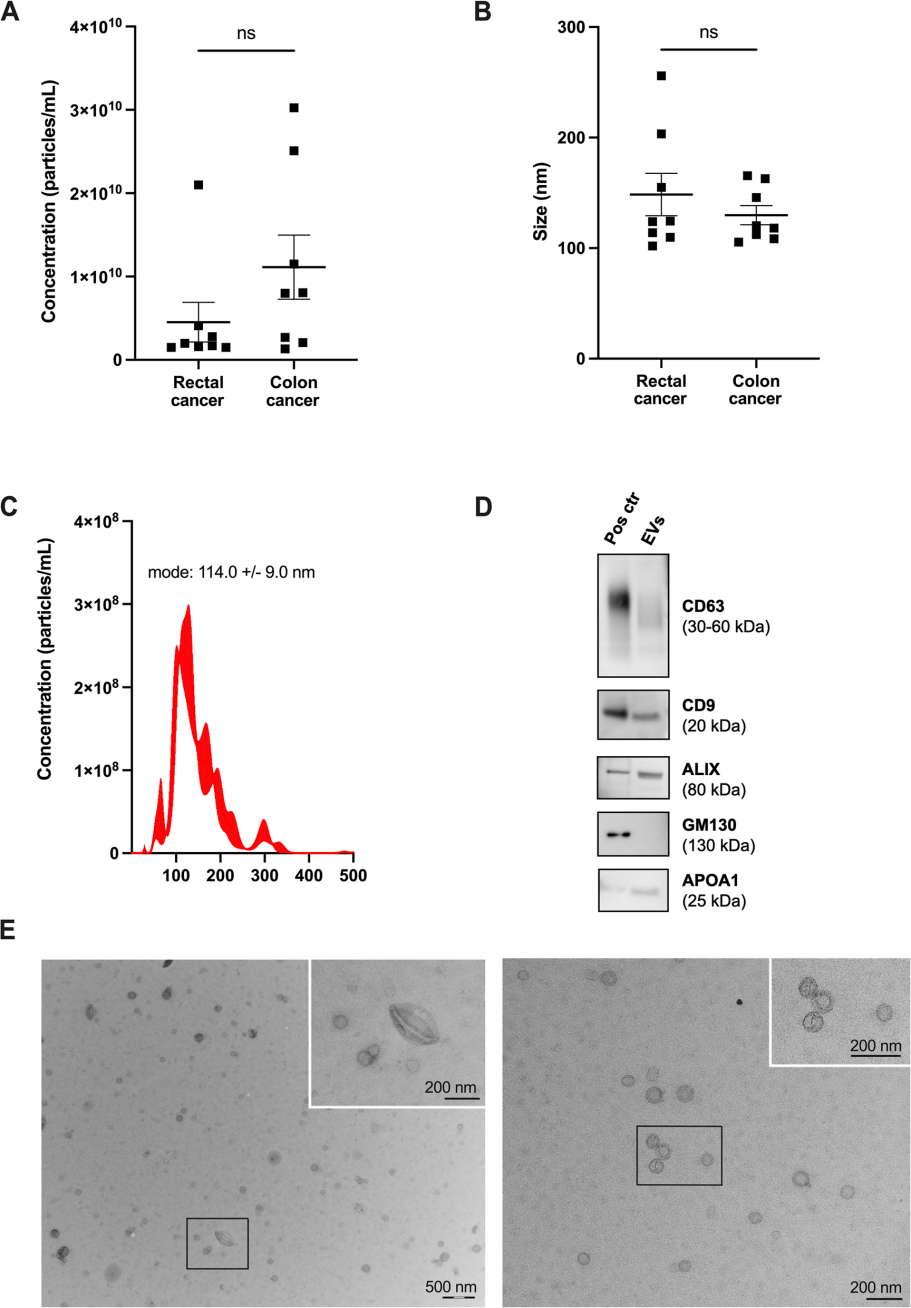
Characterization of plasma extracellular vesicles (EVs) from the colorectal cancer patients. **A** Concentration (mean ± standard error) and (**B**) mode size (mean ± standard error) of vesicles (*n* = 16) measured by Nanoparticle Tracking Analysis (NTA). The mean value of each cancer type was compared by Mann–Whitney U test (ns, not significant). **C** NTA histogram (concentration and size (mean ± standard error) for the experiment) of three combined 60-s videos from a representative EV sample. **D** Western blot images of the EV markers CD63, CD9, and ALIX and contamination markers GM130 and APOA1 expression in EVs from one patient. HCT116 colorectal cancer cells were used as positive control (Pos ctr). Full-length blots are presented in Supplementary Figure S1. **E** Transmission electron microscopy images of EVs from one patient. The positions of the zoom in panels within the wide-field views are indicated by black boxes; scale bars are 200 nm (black) and 500 nm (black and white)

Characterization of plasma EVs by NTA showed median concentration of 1.85 × 10^9 (range, 1.5 × 10^9–2.1 × 10^10) particles/ml and median mode size of 124.3 (range, 102.0–255.9) nm (Fig. 2a, b). The NTA histogram (Fig. 2c), western blot (Fig. 2d; full-length blots are presented in Supplementary Figure S1), and TEM images (Fig. 2e) from the selected patient sample confirmed vesicles of various sizes with the expected cup shape and the presence of expected EV proteins (CD63, CD9, ALIX). The heterogeneous EV sample showed absence of the contamination marker from the Golgi apparatus (GM130) but APOA1, a protein found in high-density lipoproteins, was detected. The samples were pre-treated with DNase and Proteinase to eliminate contaminating molecules adherent to the EV surface or present in plasma, with a significant reduction (19%) in total DNA concentration (Supplementary Figure S2a; paired t-test: *p* = 0.0005). To examine whether the DNase treatment might artificially generate new mtDNA variants, the samples treated with and without DNase were analyzed for damage in *MT-RNR1*, with similar damage level (Supplementary Figure S2b; paired t-test: *p* = 0.49). For the total rectal cancer cohort, plasma EVs presented twice as many mtDNA variants compared to WB and PBMCs, with the median number of 113.0 (range, 70–224; Dunnett's multiple comparison: *p* = 0.046 and *p* = 0.020, respectively; Fig. 1a).

The FFPE tumors showed significantly higher mtDNA variant number than the other tissues, with median of 327.5 (range, 167–391; Dunnett's multiple comparison: *p* < 0.0001; Fig. 1a), corresponding to approximately sevenfold the WB number. This finding was not unexpected as the FFPE-derived DNA was fragmented and the mtDNA genome was amplified in a small-amplicon format that possibly could introduce false positives. The fixation process impacts the quality of the DNA with undesirable modifications such as deamination that introduces C:G > T:A mutations, and these substitutions were associated with TVN (Pearson correlation: r = 0.93, *p* = 0.0009); hence, the FFPE-data must be considered with care because of the different pre-processing protocols. Both FFPE-tumor tissue and EVs had considerable amounts of private variants not detected in WB or PBMCs. 104 variants (4.8%) were shared among all the sample types from the rectal cancer patients and the EVs and FFPE tumors had most overlap in variants among any two types (Fig. 1c). The mtDNA variants were also analyzed for the level of heteroplasmy, as an initial investigation into the diversity of the mitochondrial genomes in the various tissues (Fig. 1e). As shown in Fig. 1g, low-level heteroplasmy (< 0.10) mtDNA variants were more frequent in the EVs than in WB and PBMCs (Repeated-Measures ANOVA: sample types < 0.0001 and patients *p* = 0.84; Dunnett's multiple comparison: *p* = 0.042 and *p* = 0.017, respectively), whereas FFPE tumor samples had increased number of low-level heteroplasmy compared to EVs (Dunnett's multiple comparison: *p* < 0.0001).

### mtDNA variant numbers and heteroplasmy – colon cancer patients

We sequenced WB, EVs, and FF tumor samples (Q30, median of 88.4%) available from eight colon cancer patients to investigate further if EVs and tumor tissue hold increased mtDNA variants. Here, all biospecimens were rapidly frozen, circumventing the effects of artificial mutations induced by formalin fixation. The median sequencing coverage depth was 12 289 × , and the proportion of reads mapping to the reference mitochondrial genome of WB, EVs, and FF tumor tissue were 99.3%, 99.7%, and 99.5%, respectively. The ratio of transversions and transitions, and GC content were comparable for WB, EVs, and FF tumors (Supplementary Table S4). When comparing the various tissue types, differences were detected between groups (Repeated-Measures ANOVA: sample types *p* = 0.0061and patients *p* = 0.24).

The colon cancer plasma EVs had median concentration of 8.03 × 10^9 (range, 1.3 × 10^9–3.0 × 10^10) particles/ml and median mode size of 119.3 (range, 105.4–165.5) nm (Fig. 2a, b). We could verify an increased total number of mtDNA variants in EVs (median 86.5; range, 54–202) compared to WB (median 58; range, 34–115) (Dunnett's multiple comparison: *p* = 0.021) as well as to FF tumor tissue (Dunnett's multiple comparison: *p* = 0.0047; Fig. 1b). 125 variants (16.3%) were shared among all sample types from the colon cancer patients, and although WB and FF tumor (median 51; range, 53–66) had similar total variant numbers, tissue-specific features appeared (Fig. 1d). As also shown in Fig. 1d, similar to the rectal cancer patient, the EVs contained extensive exclusive variants and the EVs overlapped with the tumor tissue to a higher degree than WB did. All EV mtDNA sequencing data was used to further emphasize the full mtDNA genome present inside the vesicles, represented by a circular plot (Supplementary Figure S3).

Figure 1f shows the colon cancer patients’ mtDNA variants represented as homoplasmic, heteroplasmic, and low-level heteroplasmic states. Low-level heteroplasmy variants were abundant in the plasma EVs compared to WB and FF tumor (Repeated-Measures ANOVA: sample types *p* = 0.0066 and patients *p* = 0.53; Dunnett’s multiple comparison: *p* = 0.033 and *p* = 0.0044, respectively; Fig. 1h).

Sequencing the complete mitochondrial genome in FFPE tissues necessitated multiple PCR primer sets for mtDNA amplification because the long-range PCR did not amplify successfully. In order to investigate if the different mtDNA amplification methods could explain the differences in variant number, the multi-primer method was applied on WB samples and the results compared with the original (two primer pairs) sequence data from three of the colon cancer patients. The multi-primer method (the technical quality of the sequence reads is shown in Supplementary Table S4**)** yielded an approximately tenfold increase in variant number to median 612 (range, 562–721) from 60 (range, 53–66), pointing to a bias with the use of multiple primers (Supplementary Figure S4; paired t-test: *p* = 0.0057). Amplifying the mtDNA genome in small-amplicon format can increase the risk of involving NUMTs segments that can be misinterpreted as mtDNA heteroplasmy. Indeed, a considerably increased risk of NUMTs co-amplification with the multi-primer approach was observed for the WB samples (Supplementary Table S5). The FFPE tissue had an increased chance of co-amplified NUMTs compared to the other samples, and also indicated cross-contamination (Supplementary Table S5), making FF tumor tissue more appropriate for analysis of the full mitochondrial genome.

### Distribution of variants within the mitochondrial genome

Among the mtDNA variants detected in rectal cancer patient samples, 39.6% in WB, 40.0% in PBMCs, 54.0% in plasma EVs, and 52.0% in FFPE tumors were distributed along protein-coding regions and the remaining in non-coding-regions (D-loop, rRNA, tRNA, intergenic regions). In the colon cancer patient samples, the proportions were 42.7% of the WB variants, 48.4% in EVs, and 47.8% in FF tumor. Normal-cell heteroplasmies tend to cluster within the non-coding D-loop, whereas tumor-specific somatic mutations are more evenly dispersed across both coding and non-coding regions [25]. Interestingly, for both cancer types, coding region variants versus D-loop variants was highest in EVs (4.2 for rectal cancer, 3.8 for colon cancer) and higher in tumor tissue (2.3 for both FFPE rectal cancer specimens and FF colon cancer specimens) than in WB and PBMCs (1.6 for the rectal cancer samples, 1.8 for the colon cancer samples), suggesting that plasma EVs contained molecular information towards acquisition of functional variants in their mtDNA.

**Fig. 3:**
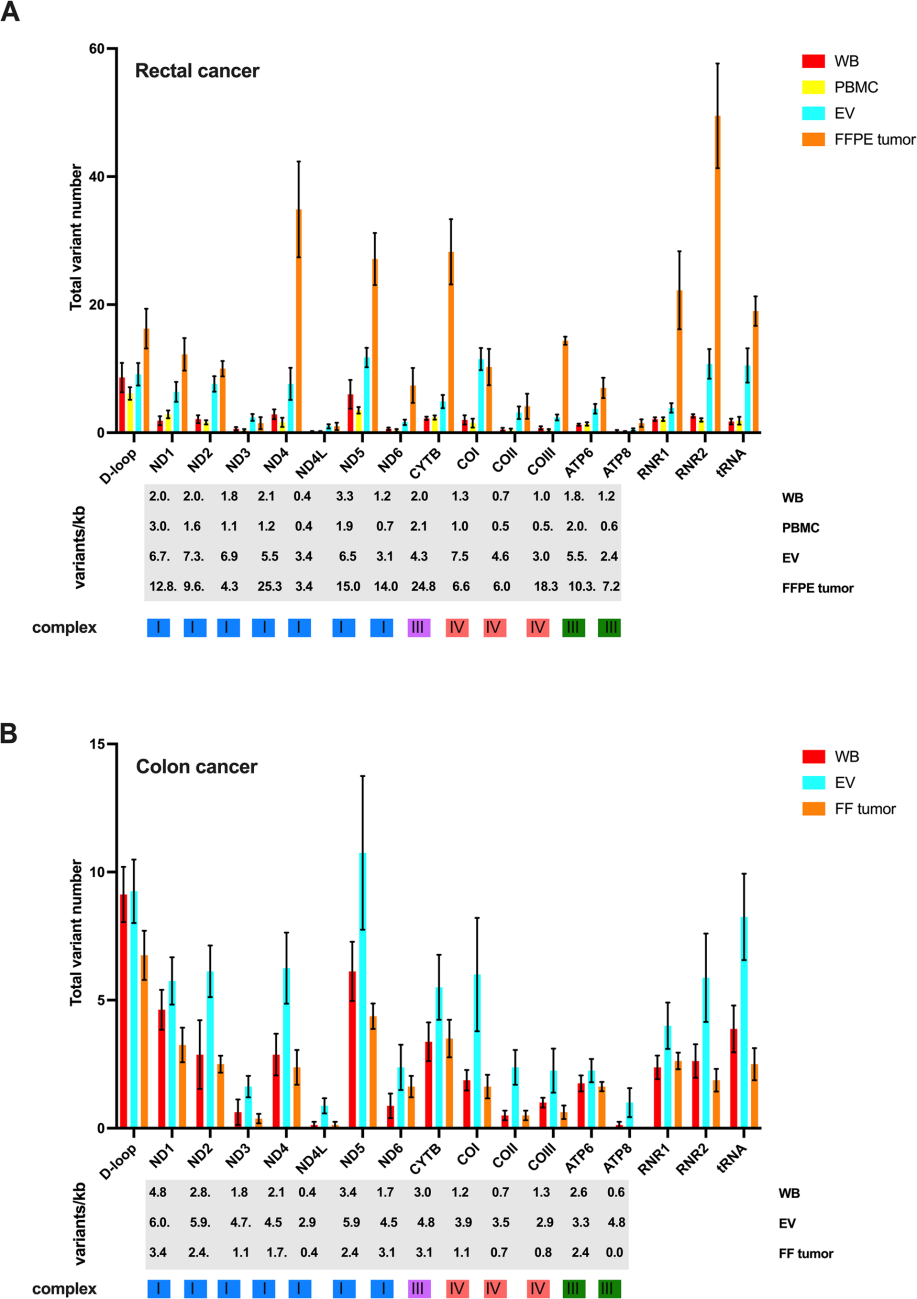
Variant numbers of the individual mitochondrial genes, including non-coding regions, in the colorectal cancer patients. Mean ± standard error of the variant number (upper panels) in each mitochondrial gene and the non-coding regions (D-loop, rRNA, tRNA) and the variant mutation rate per kilobase (kb; lower panels) are shown for each gene, as analyzed in whole blood (WB), peripheral blood mononuclear cells (PBMC), plasma extracellular vesicles (EV), and formalin-fixed paraffin-embedded (FFPE) or fresh-frozen (FF) tumor specimens from (**A**) rectal cancer patients (*n* = 8) and (**B**) colon cancer patients (*n* = 8). The mitochondrial complex of which each gene encodes a protein subunit for, is shown at the bottom

We found differences between the sample types and the various regions of the mtDNA in both the rectal (2-way ANOVA: regions *p* < 0.0001 and sample types *p* < 0.0001) and colon (2-way ANOVA: regions *p* < 0.0001 and sample types *p* = 0.038) cancer patients (Fig. 3). For the 13 mitochondrial genes, the majority showed quite similar numbers in WB and PBMCs and higher numbers in EVs and FFPE tumors from rectal cancer patients (Fig. 3a). In the colon cancer specimens (Fig. 3b), WB and FF tumor showed quite similar numbers of variants for the genes, while EVs had higher variant numbers. *MT-ND5* (NADH dehydrogenase, subunit 5 of complex I) had most variants in all samples, except in FFPE tissue with *MT-ND4* (NADH dehydrogenase, subunits 4 of complex I) as most affected. Based on mutations per kilobase (Fig. 3a, b), *MT-ND5* and *MT-ND4* were also the most affected in WB and FFPE tissue samples, respectively, from the rectal cancer patients. Generally, in plasma EVs, *MT-ND1* (NADH dehydrogenase, subunit 1 of complex I) and *MT-CO1* (cytochrome c oxidase, subunit 1 of complex IV) had most mutations per kilobase.

### Predicted variant effects on the protein structure

**Fig. 4:**
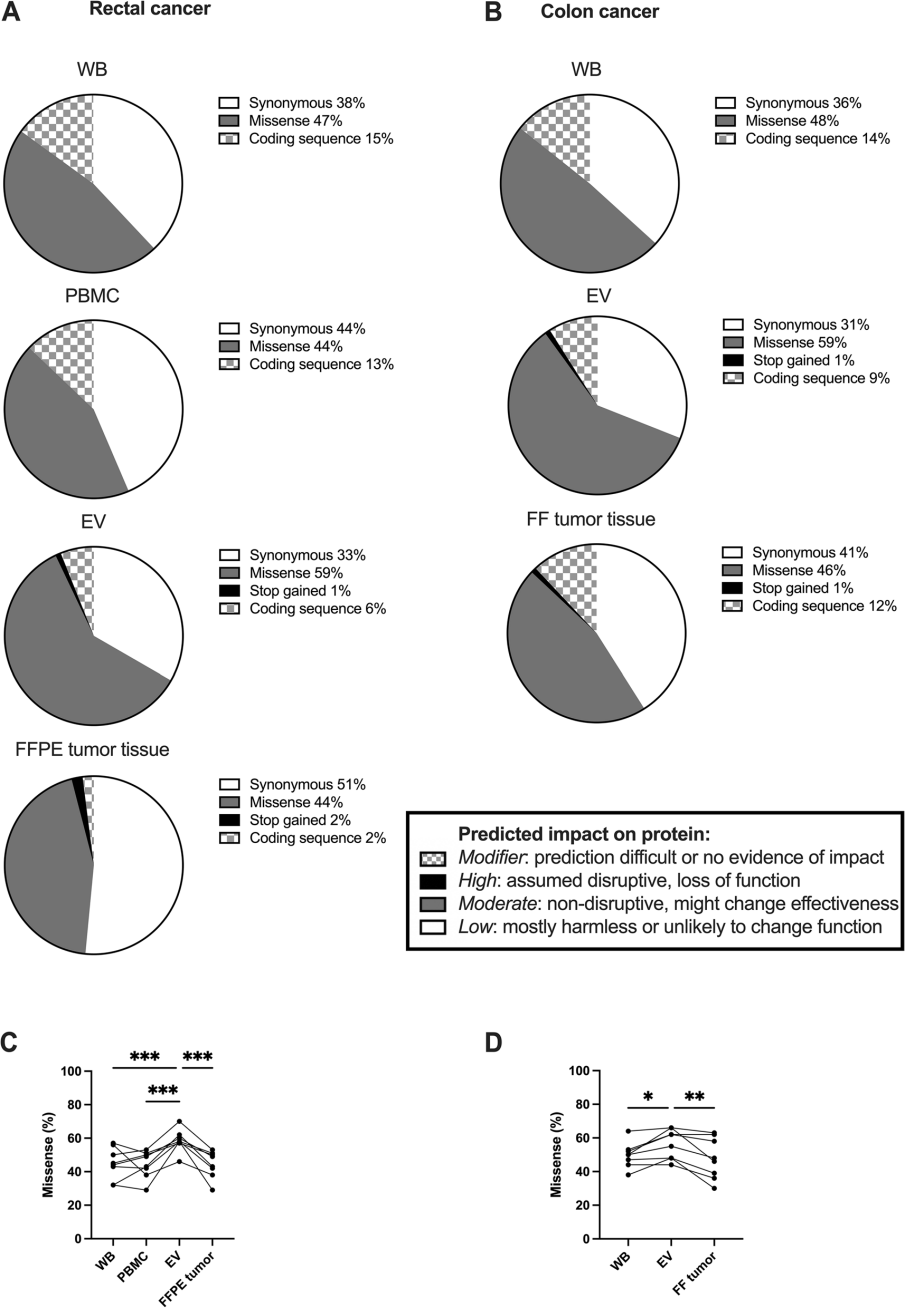
Variant effector predictions of mitochondrial DNA in the colorectal cancer patients. Distribution of mutation types in coding sequences of whole blood (WB), peripheral blood mononuclear cells (PBMC), plasma extracellular vesicles (EV), and formalin-fixed paraffin-embedded (FFPE) or fresh-frozen (FF) tumor specimens in (**A**) rectal (*n* = 8) and (**B**) colon (*n* = 8) cancer patients. Missense mutation burden (in %) of WB, PBMC, plasma EV, and FFPE tumor specimens from (**C**) rectal cancer (*n* = 8) patients and WB, plasma EV, and FF tumor specimens from (**D**) colon cancer (*n* = 8) patients. Points connected with a line represent matched patient specimens. The mean number of all variants detected in each sample type was compared to the EV number by Dunnett´s multiple comparison (*, *p* = 0.033; **, *p* = 0.0021; ***, *p* = 0.0002; ****, *p* < 0.0001)

Finally, we determined potential consequences of the variants in the coding mtDNA sequences (Fig. 4a, b) to explore whether protein structures might be affected. The tumor tissues and plasma EVs contained nonsense mutations that cause premature stop codons estimated to have high effect on the protein function (disruptive, probably causing protein truncation, loss of function, or trigging nonsense-mediated decay). These were not detected in WB and PMBCs. In addition, the EVs were more abundant in missense mutations (Fig. 4c, d) that change the amino acid and normally have moderate effects on the transcripts (non-disruptive, might change effectiveness) in both rectal (Repeated-Measures ANOVA: sample types *p* = 0.0002 and patients *p* = 0.0050; Dunnett’s multiple comparison: EVs versus WB *p* = 0.0005, EVs versus PBMCs *p* = 0.0004, EVs versus FFPE tumor *p* = 0.0004) and colon (Repeated-Measures ANOVA: sample types *p* = 0.0049 and patients *p* < 0.0001; Dunnett's multiple comparison: EVs versus WB *p* = 0.020 and EVs versus FF tumor *p* = 0.0036) cancer patients. The synonymous variants are estimated to have low effect on protein and assumed to be mostly harmless or unlikely to change protein behavior. Coding sequence variants are listed as modifiers and their predictions are difficult.

### Biological relevance of circulating mtDNA

We further investigated whether circulating mtDNA characteristics were dependent on the patient TN-status (Supplementary Figure S5). Higher degree of low-level heteroplasmy (< 0.10) mtDNA variants was observed in EVs from patients with lymph node metastasis (N1-2; Mann–Whitney U test: *p* = 0.046). Taken together, the EV mtDNA pointed towards a complex composition of secreted mtDNA with more low-level heteroplasmy and variants with potential impact on the transcripts.

## Discussion

In this study, for the first time we successfully isolated and sequenced the mtDNA cargo of plasma EVs for comparison with the mtDNA in WB, PBMCs, and tumor tissue from CRC patients. The EVs were abundant in mtDNA with more complex composition, including a higher degree of low-level heteroplasmy, compared to WB as reference. The EVs had numerous private mtDNA variants not detected in WB, PBMCs, or tumor tissue. The variants clustered in the coding regions, forming mutations with impact on the transcripts. Our data also highlights the possibility of analyzing the full mitochondrial genome of FFPE tumor tissue, but the technical requirements implied that FF tumor tissue was more expedient for the purpose. In both cases, a higher overlap of variants was detected between EVs and tumor tissue than WB and tumor tissue, suggesting that circulating EV mtDNA could be interesting to further study.

We have previously shown that plasma EVs from the rectal cancer patients, when fed to cultured human monocytes, caused monocyte transcriptional responses comprising protein binding, apoptotic mitochondrial changes, immune cell signaling, and cell growth, among other biological processes [26]. In the present study, we verified that EVs contained the intact mitochondrial genome. The total DNA concentration of the EV samples was reduced by the DNase and proteinase treatment, but it did not affect the total mtDNA variant number (data not shown) nor did it cause more mtDNA (*MT-RNR1*) damage. Overall, this suggests that the full genome is present and protected inside the vesicles.

Replication is the primary source of new mutations in mtDNA. The mutation rate observed in mtDNA is 10–17 times higher than that of the nuclear genome, and caused by the lack of histones, efficient DNA repair mechanisms, and the proximity to reactive oxygen species generated by oxydative phosphorylation [27]. Tumor cells have altered bioenergetic processes, such as increased glucose metabolism, altered calcium regulation, altered production of reactive oxygen species, or altered interorganelle interaction. These changes may result from pre-existing or de novo mutations of nuclear- or mtDNA, changes in gene copy number, or altered gene expression [5]. The plasma EV mtDNA variants were in both the coding and non-coding regions but with high protein-coding region to D-loop ratio, suggesting that EV mtDNA entails adaptive metabolic features. Of the 13 protein-coding genes, *MT-ND5* had the highest number of variants in plasma EVs from both colon and rectal cancer patients. *MT-ND5* is the most frequently mutated mitochondrial gene in cancer [28]. It is evidence for negative selection of truncation mutations in the mtDNA genes, but for some malignancies, including CRC, the opposite has been shown with suggested functional oncogenic impact of mitochondrial mutations in the initiation and clonal evolution of the cancer [28]. In our study, the EV mtDNA displayed high diversity and several distinctive variants not found in the other samples, pointing towards a possible involvement of EVs in regulation of mtDNA heterogeneity of the patients. However, since we did not sequence samples from healthy controls and EVs are secreted by all cells, we cannot exclude that heterogeneous EV mtDNA variants originate from other tissues than the cancer cells. To truly answer this, further studies are needed.

The mtDNA contains three relevant classes of phenotypes; recent germline mutations, somatic mutations, and ancient adaptive polymorphisms. These variants appear within a cell with normal mtDNA generating a mixed mitochondria-containing cytoplasm of variant and reference mtDNA, a state known as heteroplasmy [5]. Detection of low-level (< 0.10) heteroplasmy has been important for diagnosis and prognostication of mitochondrial diseases, but also in cancer and age-related research [29]. In plasma EVs from both the colon and rectal cancer patients, low-level heteroplasmy variants were frequent. In order to sequence the complete mitochondrial genome in FFPE tissues, the mtDNA amplification necessitated multiple PCR primer sets. The apparent low number of variants in FF tumor compared to FFPE tumor suggested that the tumor tissue conservation method or mtDNA amplification procedure before sequencing might have had an impact on variant detection. Additional mtDNA variants might have been generated during the tissue formalin-fixation process in the form of nucleotide modifications such as G > A and C > T transitions, which previously have been suggested as resulting artifacts using this conservation [30, 31], as well as increased co-amplification of NUMTs [32, 33]. It can be possible to reduce such sequence information by experimental or bioinformatic methods, none of which is sufficiently standardized. Of note, the mtDNA copy number per cell can vary by several orders of magnitude depending on the cell type [34]. Our current strategy did not allow us to determine whether the variants came from the enrichment of mutant mtDNA or mtDNA copy number variations.

Although the origin of the EVs is unknown, in our study they showed similarities to the tumor tissues with the presence of nonsense mutations, and could possibly be involved in tumor cell signaling to adjust their metabolic needs. However, the cell has multiple pathways to recover mtDNA and maintain mitochondrial quality, including mtDNA repair, degradation, clearance, and release. Damaged mtDNA can be removed from the cells through EVs (by means of fragmented or the intact full genome), migrasomes, or other pathways of clearance, in order to maintain cell homeostasis (reviewed in [35]). The plasma EV mtDNA was abundant in coding region missense mutations, making it tempting to speculate that functionally detrimental somatic mtDNA mutations in cells can be expelled via EVs.

Limitations to this study includes the influence of sample storage and potential mtDNA amplification bias. Experimental factors [29] and especially contaminating NUMTs, generated by the transfer of mtDNA into the nuclear genome, can complicate mtDNA sequencing analysis [32, 33]. However, the long-range targeted PCR prior to sequencing circumvented this problem to some degree [36] and highlights snap-freezing as the more suitable conservation method for mitogenome analysis of solid tissues. Another limitation is the low number of patients selected mainly based on the availability of biobank materials, hampering a more thorough investigation of the biological relevance.

## Conclusion

In conclusion, our investigations revealed that plasma EV mtDNA exhibits a high degree of diversity, suggesting involvement in CRC biology.

## Supplementary Information

**Additional file 1. Table S1. **Clinicopathological characteristics of the study patients. **Table S2**. Sequences for 12 primer sets used for PCR amplification of human mitochondrial DNA. **Table S3**. Sequences for 9 primer sets used for PCR amplification of human mitochondrial DNA. **Table S4. **Evaluation of the quality of the aligned reads. Median (range) of Q30 (in %), coverage depth, mapping rate (in %), transition/transversion (TS/VS) ratio, and GC content (in %) of the mapped reads. **Table S5.** Evaluation of potential contamination sources. Mean (range) of nuclear mitochondrial DNA (NUMTs), cross-contamination >4%, and haplogroup assignment concordance reported from Mutserve. **Supplementary Figure S1. **Full-length western blot images of plasma extracellular vesicles (EVs; 10 ng) proteins from one rectal cancer patient and HCT116 cells (Pos ctr; 5 ng). The cropped fields, representing the blots in Figure 2c and each with the respective protein, are marked with dotted lines. CD9 was reprobed after CD63, and APOA1 was reprobed after GM130. Additional bands are unspecific or previous target proteins. Fujifilm Multi Gauge V3.1 was used to analyze and adjust the brightness and contrast. **Supplementary Figure S2. **DNase and proteinase treatment of the plasma extracellular vesicles (EVs).** A**) Relative total DNA concentration of EVs treated with (+) or without (-) DNase. **B**) Digital droplet PCR analysis of mtDNA damage in the 12S ribosomal RNA gene of EVs treated with (+) or without (-) DNase. ***, *p*=0.0005 (by paired t-test). **Supplementary Figure S3. **Circular representation of the extracellular vesicle mitochondrial genomes. Variants identified in plasma extracellular vesicle samples from colon and rectal cancer patients. **Supplementary Figure S4.** Mitochondrial DNA amplification by different PCR primer strategies. Total variant number in whole blood from three colon cancer patients when using two primer pairs (WB) or a multi-primer approach (WBMulti). **, *p*=0.0057 (by paired t-test). **Supplementary Figure S5. **Circulating mitochondrial DNA characteristics and patient TN-status. Total variant number in **A)** extracellular vesicles (EVs) and **B)** whole blood (WB); low-level heteroplasmy (<0.10) variants in **C)** EVs and **D)** WB; missense mutation burden in **E)** EVs and **F)** WB, in patients according to Tumor and Node status; *, *p*=0.046 (by Mann-Whitney U test).

## Data Availability

The datasets used and analyzed in the current study, including the sequence datasets in a safe data storage facility at Akershus University Hospital, are available from the corresponding author on reasonable request and in accordance with the General Data Protection Regulation of the European Union. Transfer of data or materials will require approval from the Data Privacy Officer at Akershus University Hospital and in some occasions from the Regional Committee for Medical and Health Research Ethics of South-East Norway.

## Abbreviations

EVs: Extracellular vesicles
mtDNA: Mitochondrial DNA
WB: Whole blood
PBMC: Peripheral blood mononuclear cells
FFPE: Formalin-fixed paraffin-embedded
FF: Fresh-frozen
CRC: Colorectal cancer
NTA: Nanoparticle tracking analysis
TEM: Transmission electron microscopy
NUMTs: Nuclear mitochondrial DNA
Q30: Quality score of 30
