# Supplementary material for: Next-generation sequencing reveals mitogenome diversity in plasma extracellular vesicles from colorectal cancer patients
Source: BMC Cancer. 2023 Jul 12;23:650. doi: 10.1186/s12885-023-11092-x (PMC10337118; doi:10.1186/s12885-023-11092-x)
Supplement: Supplementary file 1 — Additional file 1. Table S1. Clinicopathological characteristics of the study patients. Table S2. Sequences for 12 primer sets used for PCR amplification of human mitochondrial DNA. Table S3. Sequences for 9 primer sets used for PCR amplification of human mitochondrial DNA. Table S4. Evaluation of the quality of the aligned reads. Median (range) of Q30 (in %), coverage depth, mapping rate (in %), transition/transversion (TS/VS) ratio, and GC content (in %) of the mapped reads. Table S5. Evaluation of potential contamination sources. Mean (range) of nuclear mitochondrial DNA (NUMTs), cross-contamination >4%, and haplogroup assignment concordance reported from Mutserve. Supplementary Figure S1. Full-length western blot images of plasma extracellular vesicles (EVs; 10 ng) proteins from one rectal cancer patient and HCT116 cells (Pos ctr; 5 ng). The cropped fields, representing the blots in Figure 2c and each with the respective protein, are marked with dotted lines. CD9 was reprobed after CD63, and APOA1 was reprobed after GM130. Additional bands are unspecific or previous target proteins. Fujifilm Multi Gauge V3.1 was used to analyze and adjust the brightness and contrast. Supplementary Figure S2. DNase and proteinase treatment of the plasma extracellular vesicles (EVs). A) Relative total DNA concentration of EVs treated with (+) or without (-) DNase. B) Digital droplet PCR analysis of mtDNA damage in the 12S ribosomal RNA gene of EVs treated with (+) or without (-) DNase. ***, p=0.0005 (by paired t-test). Supplementary Figure S3. Circular representation of the extracellular vesicle mitochondrial genomes. Variants identified in plasma extracellular vesicle samples from colon and rectal cancer patients. Supplementary Figure S4. Mitochondrial DNA amplification by different PCR primer strategies. Total variant number in whole blood from three colon cancer patients when using two primer pairs (WB) or a multi-primer approach (WBMulti). **, p=0.0057 (by paired t-test). Supp [file 12885_2023_11092_MOESM1_ESM.docx]

**SUPPLEMENTARY TABLES AND FIGURES**

**Table S1.** Clinicopathological characteristics of the study patients.

|  | Rectal cancer | Colon cancer |
| --- | --- | --- |
| **Age** | median (range) | median (range) |
| Years | 63 (45-70) | 77 (54-90) |
|  |  |  |
| **Sex** | n (%) | n (%) |
| Male | 5 (62.5) | 3 (37.5) |
| Female | 3 (37.5) | 5 (62.5) |
|  |  |  |
| **TN classification*** | n (%) | n (%) |
| T2 | 1 (12.5) | 0 (0) |
| T3 | 4 (50.0) | 8 (100) |
| T4 | 3 (37.5) | 0 (0) |
| N0 | 0 (0) | 3 (37.5) |
| N1 | 4 (50.0) | 2 (25.0) |
| N2 | 4 (50.0) | 3 (37.5) |

***** Tumor-Node classification based on radiologic (rectal cancer) and histologic (colon cancer) assessment

**Table S2**. Sequences for 12 primer sets used for PCR amplification of human mitochondrial DNA.

| Primer^a^ | Position | Primer sequence | Primer^b^ | Product  (basepairs) |
| --- | --- | --- | --- | --- |
| 2F | F361 | ACAAA GAACC CTAAC ACCAG C | 1F | 1855 |
| 7R | R2216 | TGTTG AGCTT GAACG CTTTC | 1R |  |
| 8F | F1993 | AAACC TACCG AGCCT GGTG | 2F | 1564 |
| 11R | R3557 | AGAAG AGCGA TGGTG AGAGC | 2R |  |
| 14F | F3441 | ACTAC AACCC TTCGC TGACG | 3F | 1540 |
| 18R | R4983 | GGTTT AATCC ACCTC AACTG CC | 3R |  |
| 20F | F4797 | CCCTT TCACT TCTGA GTCCC AG | 4F | 1729 |
| 24R | R6526 | ATAGT GATGC CAGCA GCTAG G | 4R |  |
| 25F | F6242 | CGCAT CTGCT ATAGT GGAGG | 5F | 2069 |
| 31R | R8311 | AAGTT AGCTT TACAG TGGGC TCTAG | 5R |  |
| 32F | F8164 | CGGTC AATGC TCTGA AATCT GTG | 6F | 1684 |
| 35R | R9848 | GAAAG TTGAG CCAAT AATGA CG | 6R |  |
| 37F | F9754 | AGTCT CCCTT CACCA TTTCC G | 7F | 1846 |
| 41R | R11600 | CTGTT TGTCG TAGGC AGATG G | 7R |  |
| 42F | F11403 | GACTC CCTAA AGCCC ATGTC G | 8F | 1720 |
| 46R | R13123 | AGCGG ATGAG TAAGA AGATT CC | 8R |  |
| 47F | F12793 | TTGCT CATCA GTTGA TGATA CG | 9F | 1595 |
| 51R | R14388 | TTAGC GATGG AGGTA GGATT GG | 9R |  |
| 52F | F14189 | ACAAA CAATG GTCAA CCAGT AAC | 10F | 1207 |
| 54R | R15396 | TTATC GGAAT GGGAG GTGAT TC | 10R |  |
| 55F | F15260 | AGTCC CACCC TCACA CGATT C | 11F | 824 |
| 56R | R16084 | CGGTT GTTGA TGGGT GAGTC | 11R |  |
| CTRLREG | F15878 | TTAA CTCC ACCA TTAG CACC | 12F | 1340 |
| CTRLREG | R649 | TTTG TTTA TGGG GTGA TGTG A | 12R |  |

^a^ Primer set number published by Levin et al. [14]

^b^ Primer set number in this study

**Table S3**. Sequences for 9 primer sets used for PCR amplification of human mitochondrial DNA.

| Primer^a^ | Position | Primer sequence | Primer^b^ | Product  (basepairs) |
| --- | --- | --- | --- | --- |
| 1F | F15 | CACCC TATTA ACCAC TCACG | 13F | 1410 |
| 4R | R1425 | AATCC ACCTT CGACC CTTAA G | 13R |  |
| 5F | F1234 | CTCAC CACCT CTTGC TCAGC | 14F | 982 |
| 7R | R2216 | TGTTG AGCTT GAACG CTTTC | 14R |  |
| 19F | F4447 | TTGGT TATAC CCTTC CCGTA C | 15F | 1435 |
| 22R | R5882 | GCTGA GTGAA GCATT GGACT G | 15R |  |
| 23F | F5700 | TAAGC ACCCT AATCA ACTGG C | 16F | 826 |
| 25R | R6526 | ATAGT GATGC CAGCA GCTAG G | 16R |  |
| 26F | F6426 | GCCAT AACCC AATAC CAAAC G | 17F | 1366 |
| 28R | R7792 | GGGCA GGATA GTTCA GACGG | 17R |  |
| 30F | F7645 | TATCA CCTTT CATGA TCACG C | 18F | 1024 |
| 32R | R8669 | CATTG TTGGG TGGTG ATTAG TCG | 18R |  |
| 37F | F9754 | AGTCT CCCTT CACCA TTTCC G | 19F | 1412 |
| 39R | R11166 | CATCG GGTGA TGATA GCCAA G | 19R |  |
| 40F | F10704 | GTCTC AATCT CCAAC ACATA TGG | 20F | 896 |
| 41R | R11600 | CTGTT TGTCG TAGGC AGATG G | 20R |  |
| 57F | F15971 | TTAAC TCCAC CATTA GCACC | 21F | 934 |
| 58R | R336 | TTAAG TGCTG TGGCC AGAAG | 21R |  |

^a^ Primer set number published by Levin et al. [14]

^b^ Primer set number in this study

**Table S4. Evaluation of the quality of the aligned reads.** Median (range) of Q30 (in %), coverage depth, mapping rate (in %), transition/transversion (TS/VS) ratio, and GC content (in %) of the mapped reads.

|  | Q30 % | Coverage (reads) | Mapping (%) | TS/TV ratio | GC (%) |
| --- | --- | --- | --- | --- | --- |
| WB | 91.4  (76.7-95.2) | 12430  (2935-27279) | 96.1  (92.0-98.7) | 8.42  (0-42.0) | 44.0  (44.0-45.0) |
| PBMC | 86.4  (84.2-87.3) | 17341  (13484-21509) | 99.0  (95.3-99.7) | 24.5  (0-50.0) | 45.0  (45.0-45.0) |
| EV | 90.7  (89.3-94.0) | 10283  (3205-46193) | 99.8  (99.6-99.8) | 20.0  (0-63.0) | 45.0  (45.0-45.0) |
| FFPE | 90.4  (89.3-90.9) | 18462  (12768-24648) | 99.4  (98.9-99.8) | 12.3  (9.74-17.7) | 44.0  (44.0-45.0) |
|  |  |  |  |  |  |
| WB | 88.6  (87.9-90.0) | 14924  (10704-19420) | 99.3  (93.2-99.6) | 6.63  (0-52.0) | 45.0  (45.0-45.0) |
| EV | 84.6  (81.2-87.7) | 10019  (5111-12385) | 99.7  (96.9-99.8) | 14.1  (2.35-31.5) | 45.0  (45.0-45.0) |
| FF | 89.8  (86.7-91.0) | 14354  (10400-20959) | 99.5  (99.4-99.7) | 9.17  (0-49.0) | 45.0  (45.0-45.0) |
| Multi-primer | 86.6  (84.1-88.0) | 15342  (15292-15637) | 98.9  (98.0-99.0) | 12.1  (9.48-16.5) | 45.0  (44.0-45.0) |

WB, whole blood; PBMC, peripheral blood mononuclear cells; EV, plasma extracellular vesicles; FFPE, formalin-fixed paraffin-embedded tumor; FF, fresh-frozen tumor; Multi-primer, WB DNA amplified with the multi-primer PCR approach.

**Table S5. Evaluation of potential contamination sources.** Mean (range) of nuclear mitochondrial DNA (NUMTs), cross-contamination >4%, and haplogroup assignment concordance reported from Mutserve.

|  | NUMTs | Cross contamination >4% | Haplogroup assignment concordance |
| --- | --- | --- | --- |
| WB | 37% (29-45) | 0/8 | 0/8 |
| PBMC | 40% (31-54) | 0/8 | 1/8 |
| EV | 37% (32-47) | 0/8 | 1/8 |
| FFPE | 61% (45-76) | 4/8 | 3/8 |
|  |  |  |  |
| WB | 35% (27-43) | 0/8 | 0/8 |
| EV | 33% (26-41) | 1/8 | 1/8 |
| FF | 35% (26-48) | 1/8 | 3/8 |
| Multi-primer | 92% (92-93) | 0/3 | 0/3 |

WB, whole blood; PBMC, peripheral blood mononuclear cells; EV, plasma extracellular vesicles; FFPE, formalin-fixed paraffin-embedded tumor; FF, fresh-frozen tumor; Multi-primer, WB DNA amplified with the multi-primer PCR approach.


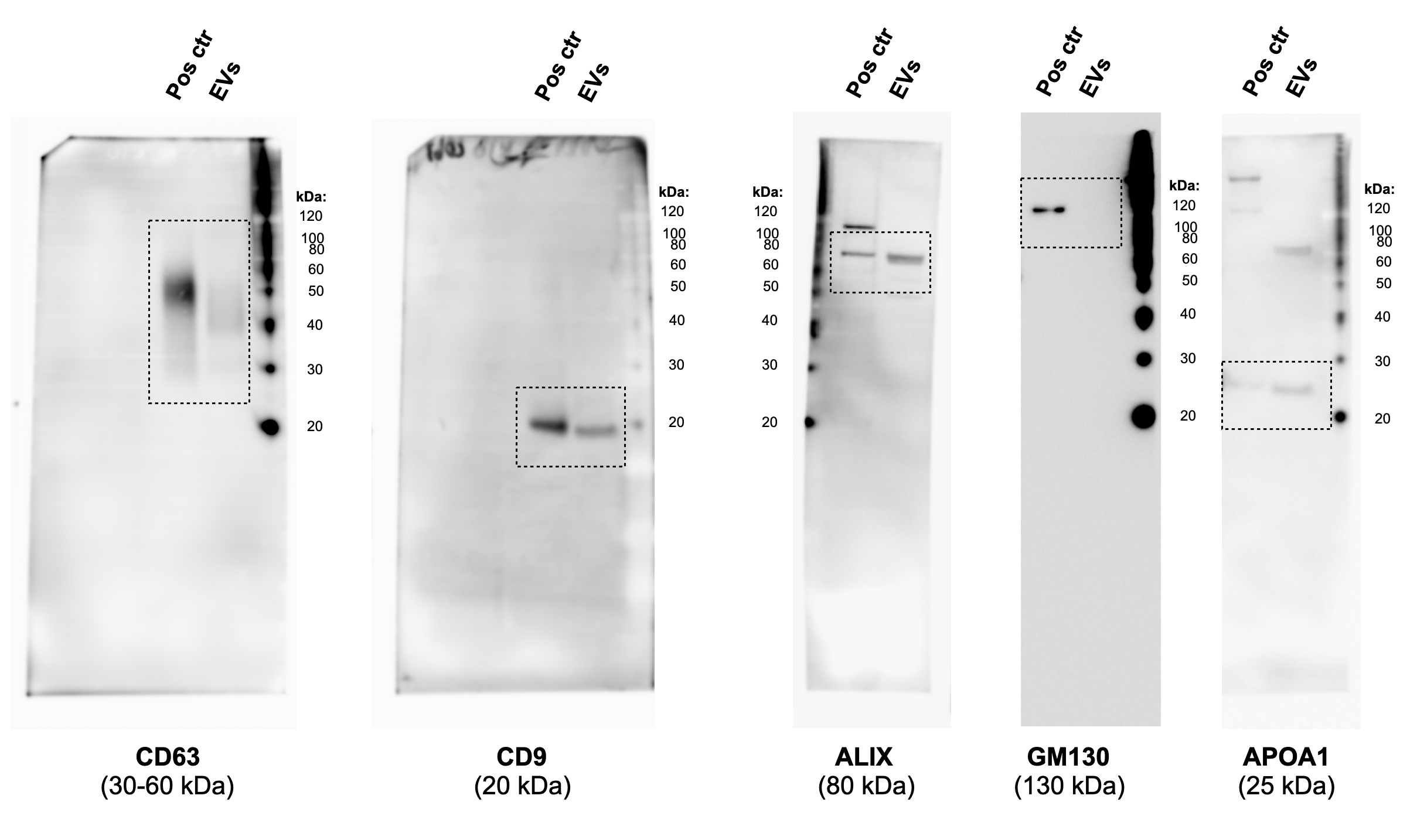
**Supplementary Figure S1. Full-length western blot images of plasma extracellular vesicles (EVs; 10 ng) proteins from one rectal cancer patient and HCT116 cells (Pos ctr; 5 ng).** The cropped fields, representing the blots in Figure 2c and each with the respective protein, are marked with dotted lines. CD9 was reprobed after CD63, and APOA1 was reprobed after GM130. Additional bands are unspecific or previous target proteins. Fujifilm Multi Gauge V3.1 was used to analyze and adjust the brightness and contrast.


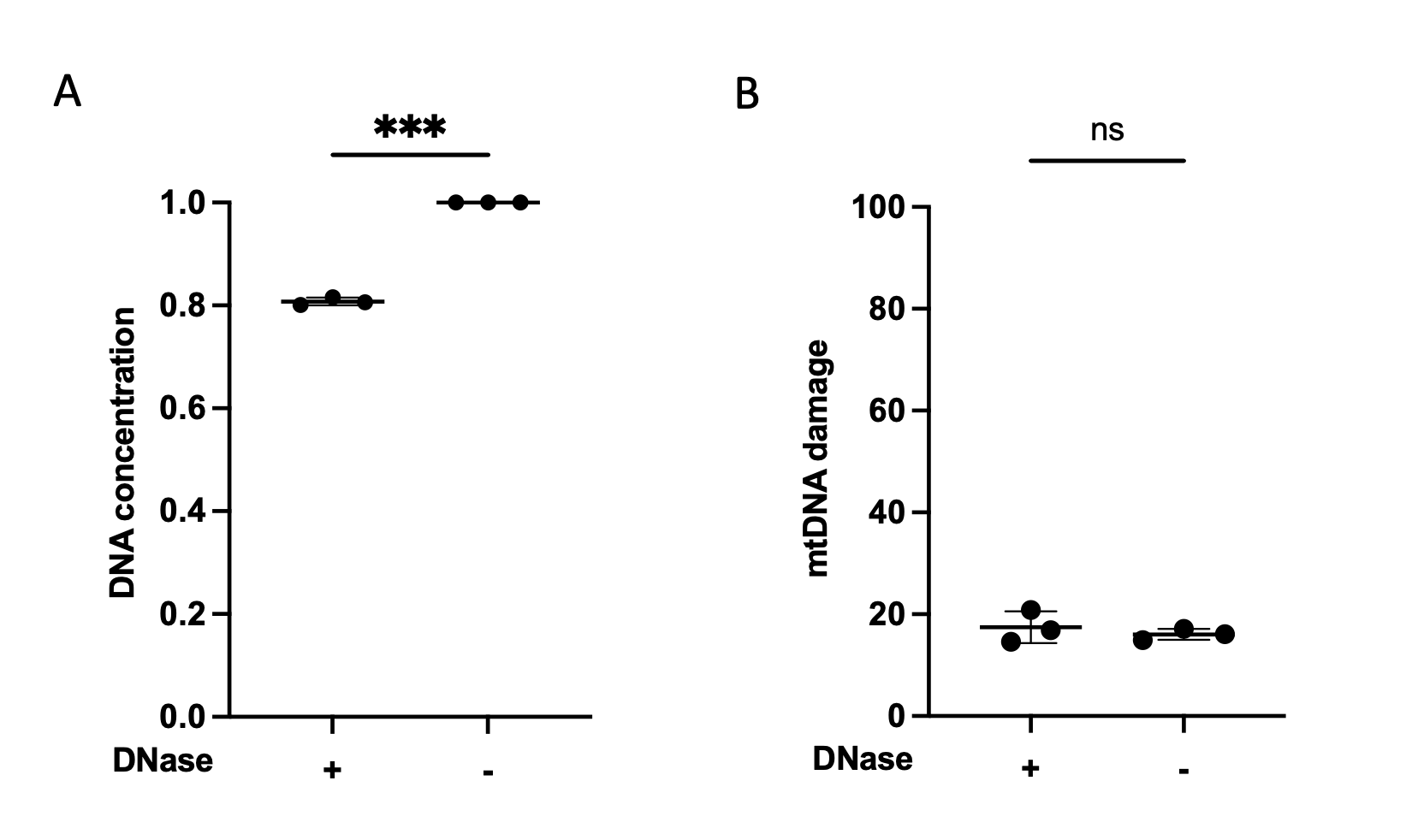


**Supplementary Figure S2. DNase and proteinase treatment of the plasma extracellular vesicles (EVs). A**) Relative total DNA concentration of EVs treated with (+) or without (-) DNase. **B**) Digital droplet PCR analysis of mtDNA damage in the 12S ribosomal RNA gene of EVs treated with (+) or without (-) DNase. ***, p=0.0005 (by paired t-test).

**
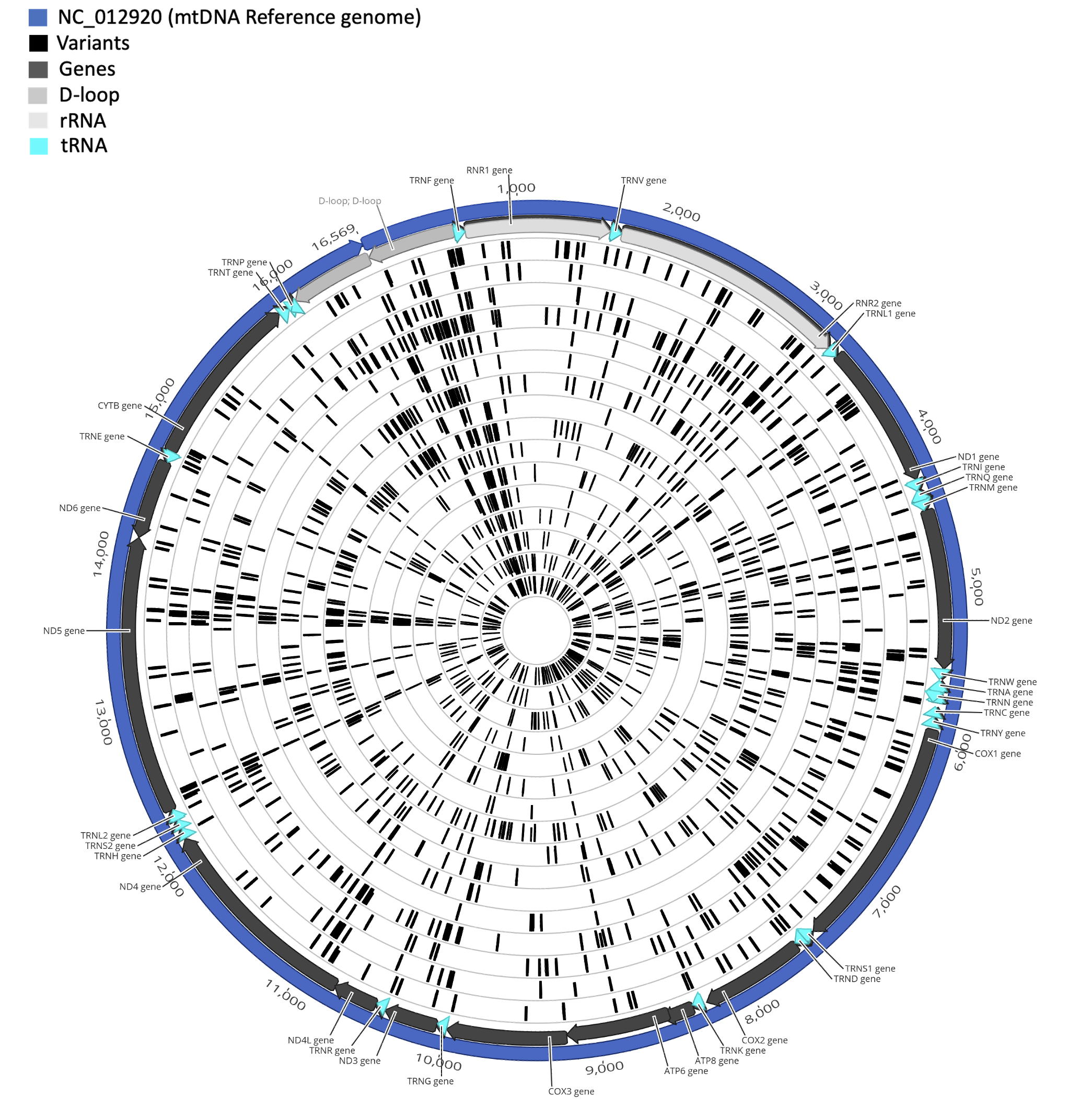
Supplementary Figure S3. Circular representation of the extracellular vesicle mitochondrial genomes**. Variants identified in plasma extracellular vesicle samples from colon and rectal cancer patients.


**Supplementary Figure S4.** **Mitochondrial DNA amplification by different PCR primer strategies**. Total variant number in whole blood from three colon cancer patients when using two primer pairs (WB) or a multi-primer approach (WBMulti). **, p=0.0057 (by paired t-test).

**
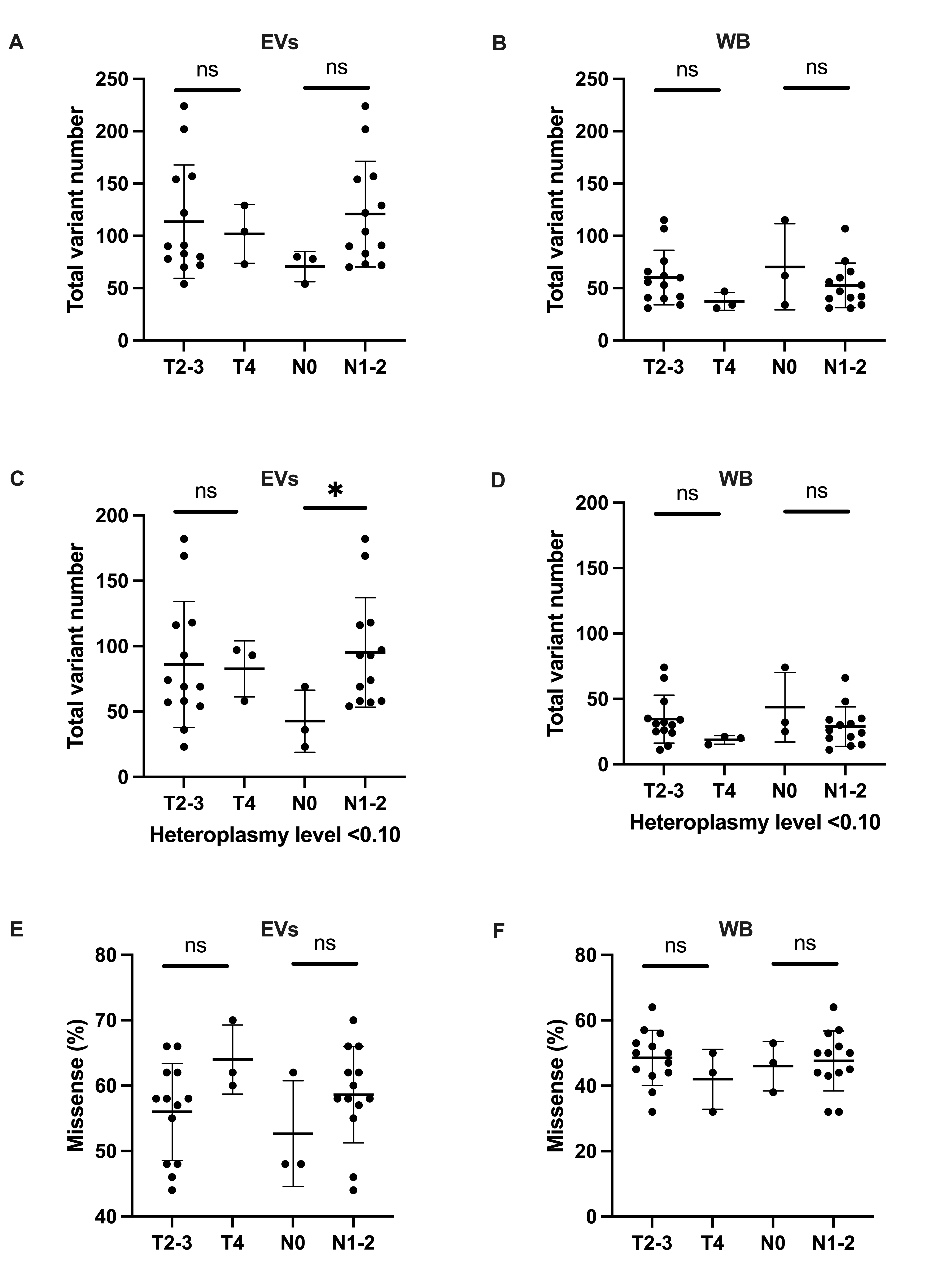

Supplementary Figure S5. Circulating mitochondrial DNA characteristics and patient TN-status.** Total variant number in **A)** extracellular vesicles (EVs) and **B)** whole blood (WB); low-level heteroplasmy (<0.10) variants in **C)** EVs and **D)** WB; missense mutation burden in **E)** EVs and **F)** WB, in patients according to Tumor and Node status; *, p=0.046 (by Mann-Whitney U test).
